# Calcium carbonate in dentistry: a bibliometric review of emerging applications and trends

**DOI:** 10.1590/1678-7757-2025-0287

**Published:** 2025-10-20

**Authors:** Andressa da Silva BARBOZA, Samira Schons de OLIVEIRA, Adriana Poli Castilho DUGAICH, Maurício Malheiros BADARÓ, Ana Paula Varela Brown MARTINS, Rafael Guerra LUND, Sheila Cristina STOLF, Juliana Silva Ribeiro de ANDRADE

**Affiliations:** 1 Universidade Federal de Santa Catarina Programa de Pós-Graduação em Odontologia Florianópolis SC Brasil Universidade Federal de Santa Catarina - UFSC, Programa de Pós-Graduação em Odontologia, Florianópolis, SC, Brasil.; 2 Universidade Federal de Santa Catarina Departamento de Odontologia Florianópolis SC Brasil Universidade Federal de Santa Catarina - UFSC, Departamento de Odontologia, Florianópolis, SC, Brasil.; 3 Universidade Federal de Pelotas Programa de Pós-Graduação em Odontologia Pelotas RS Brasil Universidade Federal de Pelotas -UFPel, Programa de Pós-Graduação em Odontologia, Pelotas, RS, Brasil.

**Keywords:** Calcium Carbonate, Dentistry, Biocompatible Materials, Dental Materials, Tooth Remineralization

## Abstract

Calcium carbonate (CaCO₃) has garnered increasing attention in dental research due to its versatile bioactive properties and expanding applications in preventive, restorative, and regenerative therapies.

**Objective:**

This study aims to comprehensively assess the bibliometric features of articles evaluating the utilization of calcium carbonate (CaCO₃) in dentistry by conducting a bibliographic search on the Web of Science databases until March 2025.

**Methodology:**

The following data were collected: number and density of citations; authorship; year, journal of publication, and impact factor; study design and theme; keywords; institution and country of origin. VOSviewer software was used to generate collaborative network maps for authors and keywords.

**Results:**

A total of 91 highly cited articles were identified, with citation counts ranging from 123 to zero. Most articles (74%) were published after 2010, with the highest prevalence in Asia (44%), especially China (10%). The most frequent study design was in vitro (55%), primarily focused on restorative dentistry (29%) and cariology (23%). The most common keywords were “Calcium Carbonate” and “Hydroxyapatite”. DeVizio W. was the most prolific author, with four publications.

**Conclusions:**

Bibliometric analysis highlights a growing interest in the application of calcium carbonate in dentistry, with a progressive increase in scientific output over the years. The findings underscore the global distribution of research and emphasize the relevance of this biomaterial in various dental specialties. This study reinforces several key points for research groups worldwide engaged in the development of innovative dental materials, providing valuable direction for future investigations, which remain limited in scope, especially regarding clinical applications and long-term outcomes.

## Graphical abstract

**Figure:**
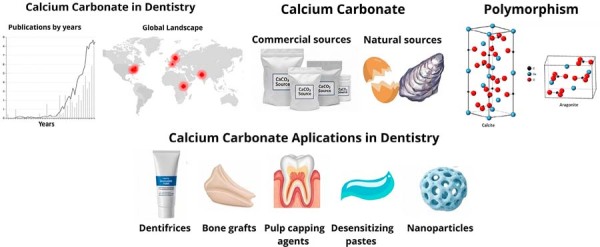

## Introduction

Calcium carbonate (CaCO₃) has emerged as a transformative biomaterial in modern dentistry, bridging the gap between fundamental material science and clinical innovation. As the most stable crystalline polymorph of calcium-based minerals, CaCO₃ shows exceptional biocompatibility, pH-modulating capacity, and osteoconductive properties—attributes that have propelled its integration into preventive, restorative, and regenerative dental therapies.^1-3^The unique capacity of the mineral to undergo phase transitions between calcite, aragonite, and vaterite forms enables precise tuning of its mechanical and dissolution kinetics, making it indispensable for applications ranging from bioactive fillers to drug-eluting scaffolds.^4-6^ Dental caries and periodontal diseases affects 3.5 billion people globally,^7^ so the multifaceted functionality of CaCO₃ addresses urgent unmet needs in oral healthcare, particularly in minimally invasive and sustainable treatment paradigms.

The renaissance of CaCO₃ in dentistry is driven by nanotechnology-enabled breakthroughs. Nano-structured CaCO₃ (nCaCO₃) demonstrates superior bioactivity compared to conventional micron-sized particles, with 40–60% higher remineralization efficiency in demineralized enamel.^8^ This enhancement stems from nCaCO₃’s increased surface area and ion release kinetics, which facilitate rapid apatite nucleation.^9^ Clinically, nCaCO₃-doped composites show greater compressive strength than commercial controls,^2,9^ while CaCO₃-infused dentifrices reduce secondary caries incidence by 22% over two-year follow-ups.^8,10,11^ Furthermore, CaCO₃’s synergy with fluoride and antimicrobial agents has yielded next-generation formulations that simultaneously disrupt biofilms, buffer acidic challenges, and promote mineral gain—a triadic mechanism critical for caries management in high-risk populations.^3,12,13^

Beyond prevention, CaCO₃ is redefining regenerative dentistry. As a scaffold component, its interconnected porosity, large surface area, and controlled degradation kinetics support angiogenesis and osteogenesis.^2,9^ The mineral’s intrinsic immunomodulatory properties further enhance healing, significantly reducing pro-inflammatory cytokines (IL-1β, TNF-α) in periodontal defects.^14^ Recent advances exploit CaCO₃ as a stimuli-responsive carrier, in which pH-triggered release of antibiotics (e.g., doxycycline) achieves localized and sustained antimicrobial activity at periodontal pockets—a paradigm shift from systemic dosing.^2,14^ Such innovations underscore the potential of CaCO₃ to revolutionize personalized and precision dentistry.

Bibliometric analysis represents a powerful tool for mapping the evolution of scientific fields, offering quantitative insights into research trends, collaborative networks, and emerging frontiers.^15^ In this context, the applications of calcium carbonate (CaCO₃) in dentistry are particularly salient, given the material’s expanding role across preventive, restorative, and regenerative therapies. This methodology can reveal critical shifts in research priorities by systematically analyzing publication patterns—from early explorations of the abrasive properties of CaCO₃ to contemporary investigations into its nanostructured formulations and bioengineered composites. Moreover, bibliometric techniques enable the identification of underserved research areas, such as the long-term clinical performance of CaCO₃-based materials or their interactions with the oral microbiome, which remain poorly characterized despite their clinical relevance.

To date, no comprehensive bibliometric assessment has been conducted to synthesize the global research landscape of CaCO₃ in dental science. This gap impedes efforts to optimize resource allocation, foster cross-disciplinary collaboration, and accelerate the translation of laboratory innovations into clinical practice. This study addresses this need by employing rigorous bibliometric methods to evaluate the scientific output related to CaCO₃ in dentistry, with particular attention to thematic evolution, geographic distribution of knowledge production, and the interplay between material science and clinical research. Specifically, it seeks to identify the most influential publications, emerging thematic areas, and global contributors, while highlighting underexplored topics such as clinical translation and long-term outcomes. By doing so, this bibliometric analysis is expected to support more informed decision-making among researchers, funding bodies, and dental biomaterials developers. Furthermore, delineating these dimensions provides a foundational framework for future investigations and highlights opportunities to bridge existing gaps between fundamental research and applied dental medicine. The findings are expected to inform strategic decision-making for researchers, funding agencies, and industry partners invested in advancing oral biomaterials.

## Methodology

### Data collection

**Figure 1:**
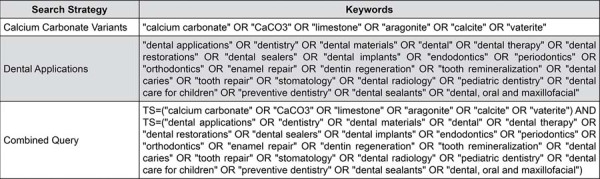
Search strategy for CaCO3 in dental applications on Web of Science (WoS).

The bibliometric review methodology followed established models proposed by Donthu, et al.^16^ (2021) and dos Anjos, et al.^17^ (2023), and the protocol was registered on the Open Science Framework (DOI: 10.17605/OSF.IO/C2TVZ). A comprehensive search was conducted in the Web of Science Core Collection (WoS-CC) database (https://www.webofscience.com) on March 29, 2025, using the institutional access provided by the Federal University of Santa Catarina. The search strategy (Figure 1) was designed to identify publications addressing calcium carbonate (CaCO₃) in dental contexts. No filters were applied regarding language or publication date. Only original research articles and review papers were included, while conference abstracts and non-dental regenerative studies were excluded.

### Data screening

**Figure 2:**
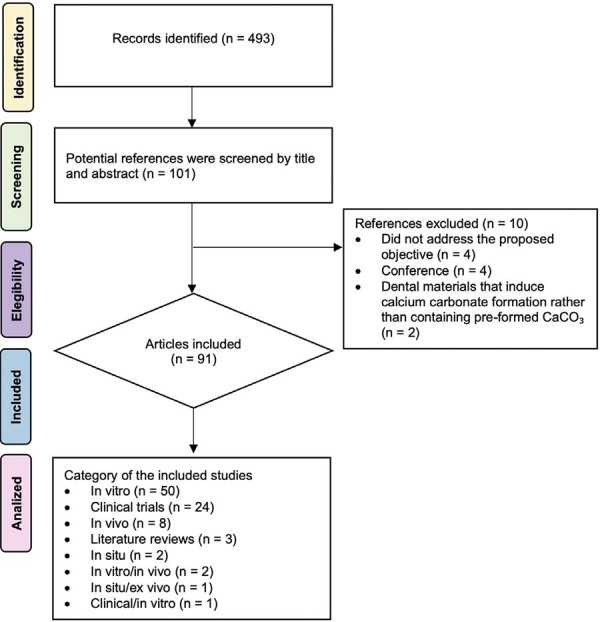
Flowchart of the search and eligibility process.

All identified articles were independently screened by two reviewers (APVBM and SCS), who evaluated titles, abstracts, and, when necessary, the full texts using the Rayyan platform for systematic reviews. The exclusion criteria were as follows: (1) studies that did not involve the application of CaCO₃ in Dentistry; and (2) materials that promoted the formation of calcium carbonate *in situ* rather than containing pre-formed CaCO₃. Studies in which CaCO₃ was part of a formulation but not explicitly mentioned in the title/abstract were systematically excluded. Discrepancies between reviewers were resolved via discussion and consensus with a third reviewer (ABS). This dual-reviewer approach was implemented to enhance screening reliability and minimize the risk of selection bias, in accordance with best practices in evidence-based reviews. Screening was implemented using the blinding function available in Rayyan (https://new.rayyan.ai/), ensuring that each reviewer was unaware of the other’s decisions during the initial selection phase and aiming to minimize selection bias and enhance objectivity. All reviewers received standardized training prior to the screening phase. Inter-rater agreement was evaluated using Cohen’s kappa coefficient (κ=0.82), indicating a strong agreement between the reviewers. Figure 2 shows a flow diagram of the study selection process.

### Data import and processing

The final dataset of included literature was imported into Rayyan to identify and remove duplicate records. Data processing involved both automated screening provided by the platform and manual verification. To ensure accuracy and reproducibility, two independent reviewers participated in the process of data extraction and classification, combining automated and manual methods to minimize errors and enhance reliability. The extracted bibliometric data included the title, authors, year and journal of publication, total number of citations and citation density (citations per year in WoS-CC), institution, country and continent of origin (based on the affiliation of the corresponding author), journal impact factor for the year 2024 (according to Journal Citation Reports), keywords, study design, and research topic.

Study designs were arranged into the following categories: *in vitro*, *in vivo*, *in situ*, combined *in vitro/in vivo, in situ/ex vivo,* clinical/*in vitro*, reviews, and clinical studies. Given the scope of the study, the included articles were also categorized according to the most prevalent dental topics: cariology, endodontics, pharmacology, dental materials, operative dentistry, implant dentistry, preventive dentistry, regenerative dentistry, restorative dentistry, periodontics, and prosthodontics.

### Bibliometric network analysis

To further explore thematic trends and author collaborations, the most frequent keywords and contributing authors were identified using the Visualization of Similarities Viewer (VOSviewer, version 1.6.17.0, The Netherlands). All data were manually verified by two independent reviewers (ASB and SSO). VOSviewer was also used to generate graphical representations of bibliometric networks, illustrating the relationships between authors (considering only those with at least two occurrences) and highlighting prominent keywords (considering only those with at least four occurrences).

Keywords in larger fonts and colored red/orange represented the most frequently used terms, whereas keywords in green/blue denoted less frequent terms. In the author network visualization (for authors with four or more occurrences), those connected and sharing the same color were part of collaborative clusters. Authors represented by larger circles had a higher number of publications.

In the network analysis, clusters consisted of closely related nodes, each identified by a specific color. The node size corresponded to the total number of publications by each co-author. Larger circles indicated more relevant terms, and highly related terms were positioned closer together. The connecting lines between terms represented the strength of their association, with thicker lines indicating stronger links. In the density map, terms with greater emphasis and intensity of color (closer to red) indicated higher occurrence or correlation, whereas those with lighter colors (yellow or green) suggested lower frequency or relevance.

### Statistical analysis

Spearman’s correlation analysis was performed to evaluate the relationship between the number of citations, journal impact factor (IF), and year of publication. Kolmogorov–Smirnov test was applied prior to the analysis to assess data normality, which indicated non-normal distributions for all variables. Given this result and the exploratory nature of the study, Spearman’s rank correlation coefficient was selected as a robust non-parametric method suitable for identifying monotonic relationships. Adjusted regression models were not applied due to the limited number of independent variables and the descriptive scope of this bibliometric analysis. All statistical procedures were performed using SPSS for Windows (version 24.0; IBM Corp).

### Language and writing assistance

Artificial intelligence tools were used during the manuscript preparation to improve the clarity and quality of the English writing. These included Grammarly^®^ (Grammarly Inc., USA), ChatGPT (OpenAI, USA), and DeepSeek (DeepSeek, China), which assisted in grammar correction, language refinement, and improvement of scientific writing style. All content was critically reviewed and validated by the authors to ensure accuracy and originality.

## Results

### Search results

The search strategy identified 493 publications. Potential articles related to calcium carbonate use in dentistry were screened by reviewing titles and abstracts for relevance to the research goals. During this process, 402 documents were excluded for not aligning with the aims of the study or for using calcium carbonate-inducing materials rather than pre-formed calcium carbonate. Additional articles were excluded due to non-dental applications of calcium carbonate. In total, 91 articles were included and categorized by study type: *in vitro* studies (n=50) were the most prevalent; followed by clinical trials (n=24); *in vivo* studies (n=8); literature reviews (n=3); and *in situ* (n=2). Four additional studies employed combined methodologies: two using *in vivo*/*in vitro* designs (n=2); one clinical/*in vitro* (n=1); and one combining *in situ/ex vivo* approaches (n=1).

### Citation analysis

**Figure 3:**
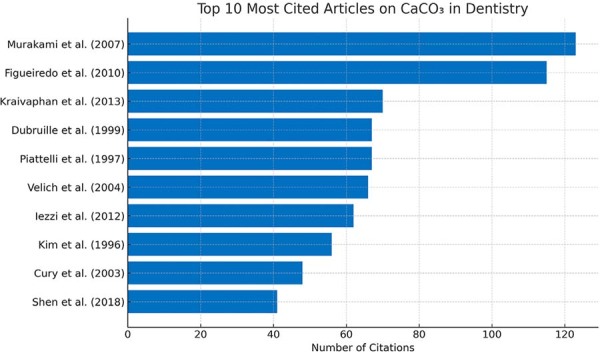
Top 10 most-cited articles on CaCO3 in dentistry based on Web of Science – Core Collection (WoS-CC).

The analyzed studies had 1,597 citations in the Web of Science Core Collection (WoS-CC) database, with citation counts ranging from 123 to 0 (Supplementary Table 1). The ten most-cited articles each accumulated over 40 citations (Figure 3). The most cited article (123 citations) was “*Physicochemical study of CaCO₃ from egg shells*”, an *in vitro* study published by Murakami, et al.^18^ (2007) in Ciência e Tecnologia de Alimentos, with an average annual citation rate of approximately seven. The second most cited article had 115 citations: “*Physicochemical Characterization of Biomaterials Commonly Used in Dentistry as Bone Substitutes - Comparison with Human Bone*”, another *in vitro* study by Figueiredo, et al.^19^ (2010) published in the Journal of Biomedical Materials Research Part B: Applied Biomaterials, showing an average of about eight citations per year. The third most cited work was Kraivaphan, et al.^10^ (2013) clinical study titled “*Two-Year Caries Clinical Study of the Efficacy of Novel Dentifrices Containing 1.5% Arginine, an Insoluble Calcium Compound and 1,450 ppm Fluoride*”, published in Caries Research, which garnered 70 citations with an average of about six annual citations.

**Table 1:** Journals that published the most on CaCO3 in dentistry.

Source title	Number of papers	Impact factor
American Journal of Dentistry	8	0.9
Caries Research	6	2.9
BMC Oral Health	4	2.6
Journal of Periodontology	3	4.2
Dental Materials Journal	3	1.9
Materials Science & Engineering C – Materials for Biological Applications	2	8.1
Ceramics International	2	5.1
Nanomaterials	2	4.4
Journal of Biomedical Materials Research Part B – Applied Biomaterials	2	3.2
International Dental Journal	2	3.2

### Publication year analysis

**Figure 4:**
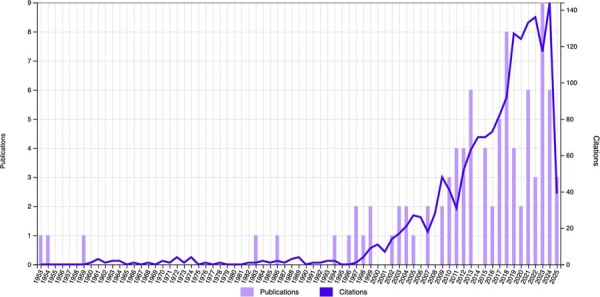
Distribution of the number of publications over the years.

The timeline of publications spans from the earliest available study—Gore^20^ (1953) article “*The role of calcium carbonate in dental caries*”—to the most recent work by Chen, et al.^14^ (May 2025) titled “*NIR-responsive CaCO₃@BMP-2/PDA nanocomposite for multifunctional therapy in periodontitis*”. The most highly cited articles were published between 1996 (n=56 citations) and 2018 (n=41 citations), collectively accounting for 42.18% of total citations (n=1,597). Figure 4 illustrates the relationship between publication year and citation frequency, demonstrating the evolving impact of CaCO₃ research in dental science.

### Contributing journals and impact factor

Table 1 shows the journals with the highest publication output on calcium carbonate in dentistry. American Journal of Dentistry led with the most publications (eight articles, 9% of total), accumulating 82 citations in WoS-CC, followed by Caries Research (six articles, 7%) with 200 citations, and BMC Oral Health (four articles, 5%) with eight citations. Regarding 2024 impact factors, the highest-ranking journals were Biomaterials (IF 12.8), International Journal of Oral Science (IF 10.8), and Materials Science & Engineering C: Materials for Biological Applications (IF 8.1).

### Study design, research topics, fields of application, and caco₃ utilization

**Figure 5:**
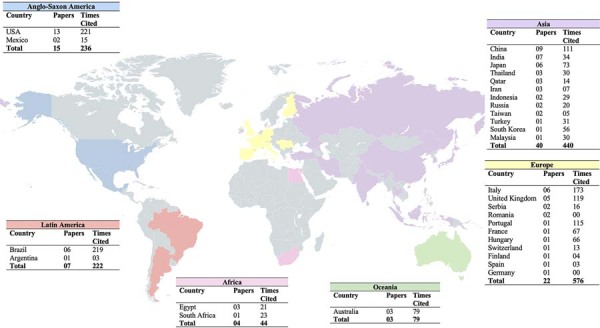
Global distribution of the origin of publications on CaCO3 in dentistry.

Most articles were *in vitro* studies (n=50, 735 citations), followed by clinical studies (n=24, 515 citations), *in vivo* studies (n=8, 169 citations), literature reviews (n=3, 68 citations), *in situ* studies (n=2, 89 citations), and combined methodologies: *in vitro/in vivo* (n=2, two citations), clinical/*in vitro* (n=1, one citation), and *in situ/ex vivo* (n=1, 18 citations) (Figure 2). Laboratory studies were predominantly conducted in China (n=9), India (n=5), Japan (n=5), and the USA (n=5) (Figure 5).

The articles covered various areas of application within dentistry, focusing on restorative dentistry (26 articles), cariology (21 articles), and implantology (18 articles). Other relevant areas included endodontics (seven articles), operative dentistry (six articles), and periodontology (five articles). Topics such as dental materials (two articles), preventive dentistry (two articles), and regenerative dentistry (two articles) were also discussed. Additionally, areas like pharmacology (one article) and prosthodontic dentistry (one article) were mentioned, albeit less frequently. This overview reflects the diversity of topics addressed in literature, with an emphasis on areas directly related to dental treatment and rehabilitation.

A quantitative distribution that highlights its versatility in dentistry can be observed based on the analysis of the frequency that CaCO₃ application was mentioned. Caries prevention was the most frequent application, recorded in 18 instances, followed by dentin hypersensitivity control, mentioned 16 times. Bone and dental regeneration had 13 occurrences, while dental implants were mentioned 12 times. Dental materials appeared nine times, and antimicrobial effects, as well as dental calculus prevention, appeared in five instances each. Other applications include studies on tooth brushing abrasion, osteogenic differentiation, mechanical properties, whitening, biomimetics, and material for pulp capping, with two articles each. Less frequent uses for calcium carbonate include use in endodontic cement, hard tissue remineralization, and as an excipient in pharmacology, with one occurrence each. Such data highlight the wide range of functions of CaCO₃, emphasizing its relevance in both functional aspects, and regenerative and protective approaches within modern dental practice.

### Caco₃-based materials and applications

Regarding the origin of the CaCO₃, 55 entries referred to commercial products, representing the most frequently reported category. Naturally derived materials were also common, with the highest frequency observed for eggshells (n=6), followed by mollusk shells (n=2), cuttlefish bone (n=1), and equisetum grass (n=1). On one occasion, the mineral was isolated and characterized from oral bacteria *Paenibacillus sp.* (n=1)*.* Synthetic or laboratory-synthesized materials were reported in eight instances. Materials with unspecified origin totaled 14, and three entries were arranged as not applicable.

Among the materials described, dentifrices predominated (31 records), confirming their widespread use in oral care products. This was followed by desensitizing pastes (eight mentions) and bone graft materials/substitutes (six mentions), highlighting their relevance in regenerative and orthopedic applications. Other reported materials included abrasives (five mentions) and specialized categories such as scaffolds, pulp capping agents, nanoparticles, and biomaterials. The diversity of CaCO₃-based materials—including composites, bioceramics, nanofibers, hydrogels, and porous structures—demonstrates the increasing sophistication and versatility of mineral-based approaches in dentistry, aligning with contemporary trends in tissue engineering and minimally invasive therapies.

### Global research landscape

The scientific literature on calcium carbonate (CaCO₃) applications in dentistry encompassed contributions from 30 countries, demonstrating the global research significance of the material. Leading contributors included the United States (13 publications, 221 citations), China (nine articles, 111 citations), Brazil (six articles, 219 citations), and India (seven articles, 34 citations). Additional participating nations comprised Japan, Italy, South Korea, Taiwan, United Kingdom, Australia, Iran, Egypt, Switzerland, Thailand, Indonesia, Germany, Mexico, Argentina, Portugal, France, Hungary, Malaysia, South Africa, Qatar, Romania, Finland, Spain, Turkey, Russia, and Serbia.

Continental analysis revealed Asia as the most productive region (40 articles, 440 citations), followed by Europe (22 articles, 576 citations), and Anglo-Saxon America (15 articles, 236 citations). Latin America contributed with seven publications, while Africa and Oceania accounted for four and three publications, respectively. Figure 5 illustrates this geographical distribution, which underscores the multinational and interdisciplinary nature of contemporary CaCO₃ research in dental applications.

### Contributing institutions

Among 145 institutions publishing on dental CaCO₃ applications, the most productive were as follows: State University of Campinas (four articles), followed by Loma Linda University, Wuhan University, Egyptian Knowledge Bank, and Colgate-Palmolive Company (three articles each). Institutions with equivalent publication counts were ranked by their cumulative citation counts in Web of Science (Table 2), highlighting both academic and industry leaders driving innovation in calcium carbonate dental research.

**Table 2:** Institutions with more publications on CaCO3 in dentistry.

Institutions	Number of articles	Number of citations
Universidade Estadual de Campinas	4	78
Colgate-Palmolive Company	3	14
Egyptian Knowledge Bank (EKB)	3	21
Loma Linda University	3	97
Wuhan University	3	67
University of Melbourne	2	56
Unilever	2	53
Kasetsart University	2	29
Voronezh State University	2	23
University of Belgrade	2	16

### Keywords

**Figure 6:**
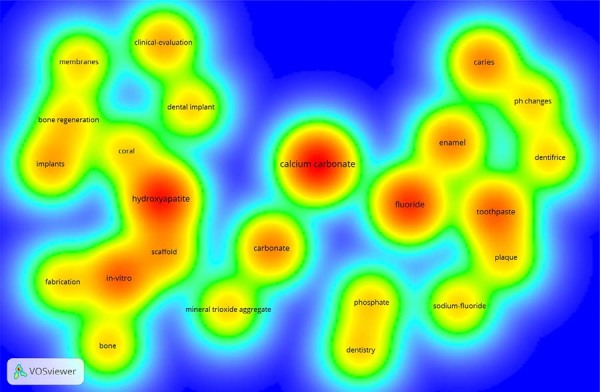
Density map of the main keywords associated with the study. A minimum of 4 occurrences was required for inclusion. Larger nodes and bold labels represent keywords with stronger co-occurrence links.

The criterion of at least several co-occurrences identified clinical evaluation (frequently cited), calcium carbonate, and hydroxyapatite as core materials of interest across studies (Figure 6). Application-focused terms such as implants, dentistry, caries, and toothpaste appeared consistently, highlighting the emphasis on regenerative and preventive dental care. Additional frequently mentioned keywords included membranes, scaffold, phosphate, and production, often linked to biomaterial development and dental tissue engineering. The presence of pH changes and dentifrice suggests a strong connection with oral environment modulation.

### Contributing authors

**Figure 7:**
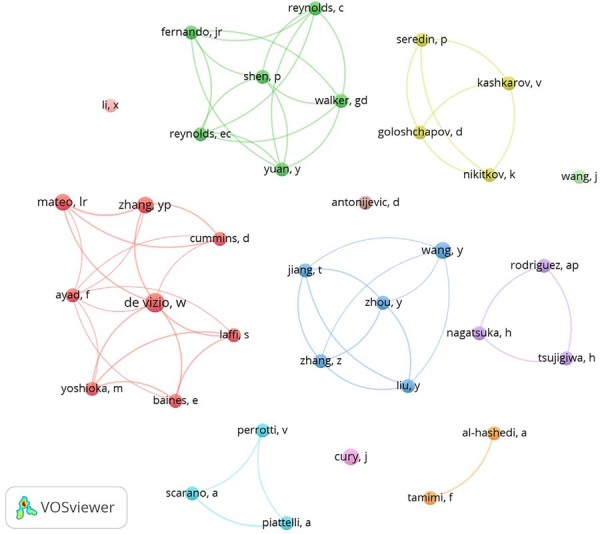
Density map of authors and their collaborative co-authorship networks.

The bibliometric analysis identified the involvement of 472 authors in the evaluated body of scientific literature. Among them, DeVizio W emerged as the most prolific contributor, with four published articles, followed by Mateo LR and Zhang YP, each with three publications. Figure 7 illustrates collaborative relationships among researchers, while Table 3 ranks the top ten authors with the highest contributions to this field of study.

**Table 3:** Authors with more publications on CaCO3 in dentistry.

Authors	Number of papers	Number of citations
Devizio W	4	104
Mateo LR	3	77
Zhang YP	3	77
Al-Hashedi A	2	3
Antonijevic D	2	16
Ayad F	2	10
Baines E	2	30
Cummins D	2	73
Fernando JR	2	64
Goloshchapov D	2	56

### Correlation analysis

Table 4 shows descriptive statistics for citation count, citation density, and year of publication. The mean number of citations per article was 17.0 (SD = 22.3), with a median of 10, while density ranged from 0.00 to 8.50 citations/year. The Shapiro–Wilk test indicated that all variables deviated significantly from a normal distribution (p<0.001), justifying the use of non-parametric correlation analysis.

**Table 4:** Descriptive statistics of bibliometric variables.

Variable	N	Mean	Median	SD	Min	Max	Shapiro-Wilk (W)	p-value
Citation count	101	17.0	10	22.3	0	123	0.716	< 0.001
Density	101	1.58	1.06	1.65	0.0	8.50	0.807	< 0.001
Publication year	101	2013	2017	13.3	1953	2025	0.714	< 0.001

Table 5 reports the Spearman’s rank correlation coefficients. Citation count showed a strong positive correlation with citation density (ρ=0.785, p<0.001), and a strong negative correlation with publication year (ρ=–0.671, p<0.001), suggesting that older studies had more time to accumulate citations. A weak but statistically significant positive correlation was observed between journal impact factor and citation count (ρ=0.203, p=0.042). Other associations were not statistically significant.

**Table 5:** Spearman’s rank correlation between bibliometric variables.

Variable Pair	Spearman’s ρ	p-value
Citation count × Density	0.785	<0.001
Citation count × Publication year	–0.671	<0.001
Citation count × Impact factor	0.203	0.042
Density × Publication year	–0.140	0.163
Density × Impact fator	0.334	<0.001
Publication year × Impact fator	0.079	0.430

## Discussion

Bibliometric reviews are essential tools for providing a comprehensive overview of a research field, enabling the identification of knowledge gaps and the proposal of new investigative directions.^21^ Such analyses guide future studies towards more specific and relevant topics within a discipline, thereby contributing to the advancement of scientific knowledge.^22^

The findings of this bibliometric analysis highlight the global distribution of scientific production related to the use of calcium carbonate (CaCO₃) in Dentistry, revealing growing interest in this multifunctional and sustainable biomaterial.^23^ CaCO₃ is a versatile biomaterial, widely available in nature, and known for its biocompatibility, bioactivity, and functionalization potential.^24^ Due to its sustainable origin, such as from oyster and mussel shells, it offers environmental value, promoting the integration of clinical innovation, waste reuse, and the redefinition of applicability within the context of green technology advancements.^25-27^ Accordingly, numerous scientific studies have investigated the use of calcium carbonate in dental applications. However, no bibliometric reviews specifically addressing this topic have been identified. Thus, this study aimed to select and evaluate the scientific profile of the most cited articles that address the use of CaCO₃ in Dentistry, to understand the broader context of this technical advancement, and to propose new directions for future developments in the field.

A thorough analysis of the main characteristics of the included articles represents a methodological strength of this bibliometric review, enhancing its quality and credibility.^16^ The decision not to impose language or publication year restrictions expanded the scope and representativeness of the sample. Furthermore, the exclusive use of the Web of Science – Core Collection (WoS-CC) database is justified by its recognized comprehensiveness and prestige in the field of bibliometric studies, as validated by previous research in Dentistry.^21,22^ Future studies could incorporate multiple databases (e.g., Scopus, Embase) to capture a broader range of studies with diverse geographic and thematic profiles. Although reliance on a single database may be considered a limitation, this strategy ensured methodological consistency and comparability with other studies, thereby strengthening the robustness of the findings.^21^

### Citation analysis

The number of citations is often used as a metric to assess the impact and relevance of a study within the scientific community.^16^ Articles with over 100 citations are considered “classics” in certain fields.^21^ Only two articles surpassed that threshold in this study (with 123 and 115 citations) while the third, fourth, and fifth ranked articles had 70, 67, and 67 citations, respectively. Compared to other bibliometric analyses in dentistry, this study revealed a relatively low number of self-citations, which enhances the credibility of the results. While self-citation can reflect productivity of the author, excessive and unjustified practices are negatively viewed for distorting impact metrics.^21^

A weak but positive correlation between journal impact factor and citation count was observed between the impact factor of journals and the number of citations received by the articles (R=0.2; p=0.042), indicating that while journal prestige may influence visibility, other factors, such as novelty, applicability, and thematic relevance, are also crucial in scientific impact.^16^ Citation density analysis showed that the most recent articles are not necessarily the most cited, suggesting that interest in CaCO₃ in Dentistry has been sustained over time, with older publications still being widely referenced.

The most cited article was titled “*Physicochemical study of CaCO₃ from egg shells*,” published by Murakami, et al.^18^ (2007), affiliated with the Federal University of Santa Catarina and the University of Joinville Region, in the journal Ciência e Tecnologia de Alimentos. The study demonstrated that calcium carbonate derived from eggshells can be a viable and sustainable alternative for dental applications, showing greater thermal stability and physicochemical properties like those of commercial control materials. The high citation count may be attributed to the practical relevance of the topic, the renewable origin of the material, and the increasing interest in environmentally responsible alternatives. Although the article does not delve specifically into dental applications, it was included in this review due to its high citation frequency within the analyzed sample.

Citation density, which is defined as the mean number of citations per year, was also analyzed to assess the longevity and sustained influence of a study over time. Murakami, et al.^18^ (2007) also stood out in this regard, showing the highest citation density. The highest citation totals were in 2007 and 2013 (154 and 146, respectively), emphasizing the importance of publications from this period in consolidating knowledge on CaCO₃ in dental contexts.

### Year of publication

The timeline of publications reveals a broad historical perspective on the role of calcium carbonate in Dentistry, spanning from the seminal 1953 article by Gore^20^(“*The role of calcium carbonate in dental caries*”) to the cutting-edge 2025 study by Chen, et al.^14^ on NIR-responsive nanocomposites for periodontitis therapy. Notably, the most influential studies were published between 1996 (n=56 citations) and 2018 (n=41 citations), collectively accounting for 42.18% of total citations (n=1597). This distribution suggests that while early research laid foundational principles, the late 1990s to late 2010s marked a peak in scientific engagement with CaCO₃ applications in dental science. The persistence of this topic over seven decades, from basic caries prevention to advanced biomaterials engineering, underscores its enduring relevance and the evolving research priorities in dental biomaterials. The recent 2025 publication further illustrates ongoing innovation in this field, particularly in therapeutic nanocomposite development.

### Contributing journals and impact factor

Among the journals most frequently publishing research on CaCO₃ in Dentistry, the American Journal of Dentistry stands out. Established in 1987 and published by the U.S.-based Mosher & Linder, Inc., this journal covers a wide range of topics, including dental therapies, restorative techniques, aesthetics, prevention, and dental materials. The leadership in the number of articles in this bibliometric review highlights its active role in disseminating advancements related to CaCO₃, particularly regarding new materials and clinical applications.

The second most prominent journal is Caries Research, internationally recognized for its focus on caries prevention, bioactive materials, innovative therapies, and evaluation of dental interventions via both laboratory and clinical studies. The frequency that these journals appear in this and other bibliometric analyses underscores their relevance to both the scientific and clinical communities, reinforcing their impact in consolidating and disseminating high-quality technical and scientific knowledge in Dentistry.

### Study design, research topics, fields of application, and uses of CaCO₃

The predominance of laboratory studies among the analyzed articles reflects a preference for experimental designs that offer greater control over variables and reproducibility. By enabling precise manipulation of experimental conditions, such studies facilitate the identification and evaluation of critical parameters associated with the performance of calcium carbonate-based materials, making their application feasible in various dental contexts. However, the predominance of *in vitro* studies could also indicate a challenge in translating these findings into clinical settings. The biocompatibility, bioactivity, low toxicity, and versatility of CaCO₃ explain the growing interest in this inorganic compound in the formulation of advanced dental products.

Despite its relevance, only three literature reviews were identified in the sample, indicating a significant gap in the field. Systematic and narrative reviews exert substantial influence on clinical practices and research directions, being considered high-level evidence sources. The scarcity of such publications may reflect the early stage of research in this area, with most studies focused on experimental design rather than clinical application. The relative novelty of this field suggests a high potential for consolidation via future reviews, particularly in identifying knowledge gaps in long-term clinical studies and regenerative applications.

Regarding application areas, restorative dentistry is the main field for CaCO₃ use, particularly in studies focused on CaCO₃ used as an abrasive agent in dentifrices, for both mechanical biofilm removal and interactions with resin-based restorations. Notable contributions were also observed in cariology, implantology, periodontology, preventive dentistry, and the development of innovative dental materials. These areas represent the ongoing search for biomaterials capable of addressing both hard and soft tissue challenges in the oral cavity.

This wide range of applications reflects the pursuit of biomaterials that act effectively on both hard and soft oral tissues. Such versatility is particularly valuable in addressing the challenges of oral regeneration, which justifies the growing interest in sustainable technologies, such as obtaining CaCO₃ from renewable natural sources or repurposed waste. Despite advances, challenges remain, such as developing structures that mimic the extracellular matrix of dental pulp and promote rapid vascularization, which are critical factors for cell viability and host cell recruitment, especially in regenerative endodontic strategies.

### Caco₃ source, and forms of use/presentation

Analysis of the selected studies revealed the diversity of calcium carbonate sources used in research, in which commercial products were the most common (n=55). This prevalence reflects the wide availability and established applicability of standardized market formulations. However, a significant number of studies also used naturally derived materials, particularly from eggshells (n=6), mollusk shells (n=2), cuttlefish bone (n=1), equisetum grass (n=1), and *Paenibacillus sp*. mineral characterization (n=1), indicating growing interest in sustainable alternatives to traditional industrial sources. The presence of CaCO₃-based compounds in two publications, alongside synthetic or laboratory-derived materials (n=8), reveals a hybrid landscape between natural and technological solutions, reflecting advances in biomaterials science.

Regarding forms of presentation and application of CaCO₃, dentifrices were markedly predominant, with 31 records, highlighting its established role as an abrasive and/or desensitizing agent in oral hygiene products. Additionally, bone grafts or substitutes (n=6) and desensitizing pastes (n=8) reinforce the role of CaCO₃ in regenerative and therapeutic contexts. Other applications include abrasive materials (n=5), scaffolds, pulp capping agents, nanoparticles, and biomaterials. Such diversity of approaches demonstrates the versatility of CaCO₃ as a functional platform in various forms and clinical settings. The growing interest in CaCO₃-based composites, bioceramics, nanofibers, and hydrogels reflects a trend towards more sustainable, bioactive, and minimally invasive solutions in contemporary dentistry.

### Global landscape

Regarding geographical distribution, the United States leads in both the number of publications (n=13) and total citations (n=221), reflecting its consolidated position as a global hub of research and technological innovation. China ranks second in both publications (n=9) and citations (n=111), due to its increasing investment in science and technology, the strengthening of its universities, and incentives for the internationalization of scientific output. Brazil also contributes significantly to this field, with results in both publications and citations. These countries host centers of excellence, robust infrastructure, and well-established collaborative networks, all of which contribute to high scientific productivity and international visibility.

Among the ten most cited articles in the analyzed sample, two were written by Brazilian researchers, including the top-ranked publication, while the United States ranks second. This result reflects the quality, the impact, and the growing relevance of Brazilian studies in the global discourse on sustainable biomaterials. The contribution of Brazilian research highlights emerging strengths in sustainable innovations, particularly the use of CaCO₃ derived from renewable sources.

### Contributing institutions

The institutional contribution analysis revealed a globally distributed research effort, with 145 institutions publishing on CaCO₃ applications in dentistry. Table 2 shows that the most productive institutions were led by the State University of Campinas (four articles), followed by Colgate-Palmolive Company, Egyptian Knowledge Bank, Loma Linda University, and Wuhan University (three articles each). Notably, the tiebreaker of cumulative Web of Science citations highlights the competitive research output among the leading institutions. The prominence of academic institutions alongside industry players (Colgate-Palmolive) reflects the translational nature of CaCO₃ research, bridging fundamental science and commercial applications in dental care. This global institutional diversity reflects the broad interest and the growing collaborations between academia and industry. This distribution suggests both broad international interest and concentrated expertise hubs driving innovation in calcium carbonate applications, from biomaterials to preventive dentistry formulations. The presence of multiple institutions with equivalent publication numbers (three articles) underscores the balanced global contribution to this field, while citation metrics reveal variations in research impact among these productive centers.

### Keywords

The identification of “calcium carbonate” and “hydroxyapatite” as core terms was expected as the most prevalent keywords, given their widespread use in dental biomaterials research. These compounds are frequently explored for their biocompatibility and potential in regenerative applications. The co-occurrence of clinically oriented terms, such as “in-vitro”, “fluoride”, “toothpaste”, and “enamel,” underscores the translational focus of these studies, particularly in preventive and restorative dentistry. The presence of “pH changes” and “dentifrice” further emphasizes the role of these materials in modulating the oral environment and maintaining homeostasis. The use of VOSviewer to create visual maps of keyword co-occurrence reflects its utility in exploring thematic structures and research trends in the field.

### Contributing authors

The bibliometric analysis revealed a collaborative research landscape involving 472 authors, who had distinct patterns in productivity and impact. DeVizio W emerged as the most prolific contributor (four articles), followed by Mateo LR and Zhang YP (three articles each), all affiliated with Colgate-Palmolive Company. Their clinical studies primarily investigated CaCO₃ in dentifrices, focusing on caries prevention, extrinsic stain removal, and gingivitis management. One study reported no significant improvement in supragingival calculus or gingivitis reduction, whereas the others highlighted the efficacy of CaCO₃ in stain removal and its potential role in caries prevention, underscoring its dual utility in aesthetic and preventive dentistry.

Notably, the most cited authors were Piatelli A and Scarano A (129 citations each), whose clinical research demonstrated the success of CaCO₃ as a bone substitute in pre-implant procedures and maxillary sinus augmentation, which emphasizes its osteogenic potential. This dichotomy in research focus, between therapeutic applications (bone regeneration) and preventive/aesthetic uses (dentifrices), reflects the versatility of the mineral in dental science. The collaboration network (Figure 7) and ranking of top contributors (Table 3) further reveal how institutional expertise (e.g., Colgate-Palmolive) and clinical research groups drive distinct yet complementary advancements. Together, these findings illustrate broad applicability of CaCO₃, from foundational biomaterial science to translational clinical outcomes, while highlighting gaps that warrant further investigation, such as inconsistent gingivitis results.

### Limitations and future directions

To contextualize these findings, it is important to acknowledge several limitations. First, the analysis was restricted to a single database (Web of Science Core Collection), which may limit the generalizability of the results. The inclusion of additional databases, such as Scopus, Embase, or regional sources, could capture studies with different thematic or geographic profiles. Second, although relevant associations were identified using Spearman’s rank correlation, the study did not employ multivariate regression models that could provide deeper insights into the factors influencing citation impact. Third, the process is still subject to interpretation bias, even though blind and independent screening was applied. Clearer reporting on reviewer calibration could strengthen the reliability of future studies, despite the high inter-rate agreement. Another limitation is the exclusion of studies in which CaCO₃ was present in the formulation but not explicitly mentioned in the title, abstract, or keywords—potentially omitting relevant clinical trials, especially those involving dentifrices and desensitizing products. Future bibliometric reviews should adopt broader search strategies, to better reflect the clinical and commercial relevance of CaCO₃ in dentistry, including full-text screening.

Moreover, future research should address gaps such as the limited number of long-term clinical studies, systematic reviews, and investigations focused on regenerative applications. The growing interest in sustainability also points to the need for further studies on biogenic and waste-derived sources of CaCO₃. Expanding keyword structures, using multiple databases, analyzing funding sources, and examining citation contexts are recommended strategies to enhance the scope and impact of future bibliometric analyses in this field.

## Conclusions

This bibliometric analysis highlights a growing interest in the application of calcium carbonate in dentistry, with a progressive increase in scientific output over the years. The findings underscore the global distribution of research and emphasize the relevance of this biomaterial in various dental specialties. This study reinforces several key points for research groups worldwide engaged in the development of innovative dental materials, providing valuable direction for future investigations, which remain limited in scope, especially regarding clinical applications and long-term outcomes, and the integration of bibliometric variables such as study design, author networks, and funding sources. Future research would benefit from the inclusion of multiple bibliographic databases, the application of advanced techniques (e.g., altmetrics, citation trajectory analysis), and the expansion of variable sets to support a more comprehensive understanding of knowledge dynamics in this field.
